# Associations between helminth infection status and the composition and concentration of fecal bile acids in school-age children in Uganda

**DOI:** 10.1038/s41598-025-11170-z

**Published:** 2025-07-15

**Authors:** Natasha J. Norton, Chloe M. M. Hand, Candia Rowel, Moses Adriko, Pengfei Cai, Donald P. McManus, Thomas G. Egwang, Lisa A. Reynolds

**Affiliations:** 1 Department of Biochemistry and Microbiology, Faculty of Science, University of Victoria, Victoria, BC Canada; 2 Vector Borne and Neglected Tropical Disease Control Division, Ministry of Health, P.O Box 1661, Kampala, Uganda; 3 Molecular Parasitology Laboratory, QIMR Berghofer Medical Research Institute, Brisbane, Australia; 4 School of Biomedical Sciences, The University of Queensland, Brisbane, Australia; 5 Department of Parasitology and Immunology, Med Biotech Laboratories, Kampala, Uganda

**Keywords:** Helminth infections, Bile acids, Fecal metabolites, Biological sex, School-age children, Metabolomics, Epidemiology, Parasitology, Parasite host response

## Abstract

Over 1 billion people globally are infected with helminths, and understanding the impact of these infections on human health is crucial for further developing effective interventions. We investigated potential associations between helminth infection status and the abundance of fecal bile acids: a group of metabolites known to impact gut physiology and function and have immunomodulatory capabilities. Fecal samples were collected from school-age children in Uganda and used to determine helminth infection status (Kato-Katz technique) and to quantify the fecal bile acid pool (UPLC-MRM/MS). We found that helminth infection status was associated with changes to the fecal bile acid pool and that these differences were dependent on the biological sex of study participants. Females who were coinfected with schistosomes and hookworms had higher levels of unconjugated secondary bile acids than helminth-negative individuals. In males, no significant associations were detected between helminth infection status and levels of unconjugated secondary bile acids, however, there were reduced levels of some species of conjugated primary bile acids in schistosome-infected individuals compared to helminth-negative individuals. Further research into the specific mechanisms underlying these associations and the functional consequences of bile acid perturbations during helminth infection may provide valuable insights into the pathophysiology of helminth infections.

**Supplementary Information:**

The online version contains supplementary material available at 10.1038/s41598-025-11170-z.

## Introduction

Helminth infections caused by schistosomes and soil-transmitted helminths remain a significant contributor to human morbidity, with approximately one fifth of the global population infected^1^. These infections disproportionately impact resource-constrained communities, particularly where sanitation measures are inadequate^2^. The morbidities resulting from chronic and high-burden helminth infections are especially notable in children and can have deleterious effects on cognitive development and physical growth, as well as causing other intestinal and liver complications, depending on the infecting species^3^. Helminth infections impact host physiology through a myriad of mechanisms, including causing physical damage, stimulating/eliciting host immune responses^4^ and disruptions to the host microbiome^5^. These interactions can result in alterations to the concentrations and availability of small molecule metabolites in the host, which may mediate further impacts on host health^6^.

Bile acids are a group of host-derived metabolites which aid with lipid absorption, shape intestinal microbial communities, and act as signaling molecules to regulate many physiological processes including immune function^7^. In humans, two primary bile acids are produced from cholesterol in the liver: cholic acid (CA) and chenodeoxycholic acid (CDCA). These primary bile acids are conjugated to an amino acid (glycine or taurine) before they are stored in the gall bladder as a component of bile and then deposited into the proximal small intestine after food consumption. Here, the glycine/taurine-conjugated primary bile acids can be deconjugated by the intestinal microbiota and then modified into a broad range of secondary bile acids, including lithocholic acid and deoxycholic acid, which are CDCA and CA derivatives respectively^8^. While the majority of bile acids are reabsorbed into the blood from the intestinal tract to be recycled in the liver, a small fraction escape reabsorption to be excreted in the feces^9^.

Helminths that infect humans have the potential to disrupt the regulation and composition of the host bile acid pool. Intestine-dwelling helminths such as *Ascaris* (a roundworm) and hookworms will be directly exposed to the intestinal bile acid pool allowing for interactions with bile acids within the gut, and some schistosome species (including *Schistosoma mansoni*) can cause major pathology in the liver, the site of bile acid synthesis. Further, helminth infections can disrupt the host bacterial microbiota^10^, which is a major regulator of the host bile acid pool^8^. Previous work has begun to examine how the bile acid pool may be altered during helminth infection: in humans, *Schistosoma japonicum* infection has been associated with modifications to the bile acid pool in sera^11^ and in mice, pathway analyses of metabolites detected in the feces following infection with either *Schistosoma *species *mansoni*, *japonicum*, or *mekongi* suggested disruptions to bile acid synthesis during infection^12^. Mice infected with the strictly enteric roundworm *Heligmosomoides polygyrus bakeri* have a disrupted bile acid pool in the region of the proximal small intestine where these worms reside^13^. Considering this, we set out to examine whether helminth infections in humans were associated with disruptions to the fecal bile acid pool.

Different helminth species dominate within particular regions of Uganda^14^. Here, we analyzed the composition of the fecal bile acid pool in school-aged children from two districts in Uganda (Mayuge and Kabale) using targeted ultra-high-performance liquid chromatography-mass spectrometry (UHPLC-MS/MRM). Study participants had varying helminth infection statuses, allowing us to examine associations between the fecal bile acid pool and infections with *Schistosoma*, *Ascaris*, hookworm, or coinfections with *Schistosoma* and hookworm. Our results revealed associations between helminth infection status and disruptions to the fecal bile acid pool. Further, we noted that associations between helminth infection status and fecal bile acid pool disruptions were not consistent between female and male study participants, suggesting that biological sex may be a variable influencing how helminth infection impacts the bile acid pool. To our knowledge, this study is the first to examine associations between helminth infection status and the fecal bile acid pool in people.

## Methods

### Ethics, participant selection and sample collection

This study took place as part of a wider program aiming to develop novel diagnostics for schistosomiasis^15^. The study protocol, participant consent and assent forms were approved by the Vector Control Division Research Ethics Committee, Kampala, Uganda, application #VCDREC160. This study was conducted with the approval of the QIMR Berghofer Medical Research Institute (QIMRB) Human Ethics Committee under the project #P3700. The Human Research Ethics Board at the University of Victoria, Canada, approved the use and analysis of samples obtained from this study at the University of Victoria through application #22-0616. The teachers in schools and community leaders within sampled regions were informed of the study, study information was given to those interested in participating, informed consent from adults was obtained, and assent for the children involved in the study was given by their guardians. Participant biological sex was self-reported by adults and reported by schoolteachers for children. Participants were given instructions as to how to self-collect fecal samples: participants passed feces onto paper placed on latrine floor and the fecal sample was then transferred into a labelled stool container. Fecal samples were collected from each participant over a sequential two-day period, with all sampling occurring between September and October 2022. Those samples selected for bile acid analyses were from school-aged children ranging between 5 and 17 years of age. We only included samples for bile acid analyses if there were ≥ 3 participants in the study that were of the same biological sex and with the same helminth infection status. Helminth infection status of each study participant was classed as either (1) helminth-negative, (2) *Schistosoma*-infected, (3) hookworm-infected, (4) *Schistosoma* and hookworm co-infected, or (5) *Ascaris*-infected (S1 Data). We present analyses of fecal bile acid concentrations from 87 study participants.

### Kato-Katz diagnostic technique to determine helminth infection status

Helminth infection status was determined on both sequential days of fecal collection for each participant using the Kato-Katz diagnostic technique. In brief, the stool sample was sieved, and 41.7 mg of the sample was placed in the center of a microscope slide and smeared using a cellophane coverslip that had been soaked overnight in a 5:1:4 solution of glycerine:3% malachite green:water. Helminth infection status was determined based on egg morphology. The number of helminth eggs detectable in each sample was determined in triplicate using a light microscope under a 10X objective lens, and the number of eggs per gram of feces was calculated by multiplying the average number of eggs present in each smear by 24. Participants with no detectable helminth eggs on either collection day were classed as negative for helminth infection. If a particular helminth species was detected on only one of the two sampling days, the individual was classed as positive for that particular helminth species. Raw helminth egg count data from both sampling days is provided in S1 Data.

### Fecal sample storage and transport for bile acid analysis

An aliquot of the fecal sample collected on day 2 of the collection process for each individual was put into a stool storage container and immediately stored on dry ice. At the end of the day, samples were stored in − 20 °C freezers in regional health care centers. Samples were transported on dry ice to Kampala, Uganda and stored at − 20 °C until transportation to Victoria, Canada. Samples were shipped on dry ice and upon arrival at the University of Victoria, were stored at − 20 °C until mass spectrometry analysis. Bile acids are relatively stable, and previous work has shown no significant changes in concentrations in primary and most secondary unconjugated and conjugated bile acids after months of storage at − 18 °C^16^.

### Bile acid quantification

Bile acid quantification was conducted by Dr. Jun Han at the University of Victoria Genome BC Proteomics Center. Samples were analyzed on an Agilent 1290 ultra high-performance liquid chromatography (UHPLC) system coupled to a 6495B Agilent QQQ mass spectrometer for targeted mass spectrometry (MS) analysis. The MS instrument was operated in the multiple-reaction monitoring (MRM) mode with negative-ion detection. Specific methodologies and techniques used in MS analysis are described in Han et al.^17^.

A standard mixture containing all targets (65 bile acids and 2 bile acid precursors; listed in S1 Data) was dissolved in 40% acetonitrile at 10 nmol/mL and serial dilutions of the standard mixture were created by diluting in 40% acetonitrile. 100 μL of each standard was mixed with 100 μL of an internal standard solution containing 14 D-labeled bile acids. 10 μL of each solution was injected to run ultra-performance liquid chromatography-electrospray negative ionization-tandem mass spectrometry (UPLC-(-)ESI-MS/MS) with MRM. Linear-regression calibration curves were created between analyte-to-internal standard peak area ratios versus molar concentrations (nmol/mL).

Fecal samples were weighed to record their wet mass and were then lyophilized and weighed again to obtain dry mass. Seventy % acetonitrile was added to each lyophilized samples to obtain a concentration of 25 μL/mg of dry fecal mass, and two 3-mm metal beads were added to each sample. Samples were homogenized at a shaking frequency of 30 Hz for 1 min three times on a MM 400 mill mixer, followed by 2 min of sonication in a water bath. Samples were centrifuged at 21,000 × g at 10 °C for 10 min for clarification, and the supernatant was diluted 10 times with 40% acetonitrile. A mixture of 100 μL of each diluted sample solution and internal standard solution was prepared and 10 μL was injected for UPLC-MRM/MS analysis. The concentrations of detected bile acids were calculated from the internal standard-calibration, linear-regression calibration curves of individual bile acids.

### Statistical analyses

GraphPad Prism (v. 10.2.3) software was used for all statistical analyses. Data sets were evaluated for normality using Shapiro–Wilk tests, and given that not all data sets were normally distributed statistical tests that did not make assumptions about normality were used throughout. Statistical tests used are indicated in Figure Legends, and full details of statistical comparisons for each Figure are included in S1 Data. Graphs present either total bile acid concentrations (combined concentrations of all detected bile acids), subcategories of bile acids (combined concentrations of individual bile acids that fall in specific subcategories), or individual bile acid concentrations. A list of individual bile acids that fall into each of the subcategories of bile acids that we present can be found in S1 Data. In general, where comparisons between bile acid concentrations between only two experimental groups were being made, Mann–Whitney tests were used, with a *p* ≤ 0.05 considered statistically significant. Where more than two experimental groups (helminth infection statuses) were being compared, Kruskal–Wallis tests were used followed by Dunn’s multiple comparison tests, with comparisons between the helminth-negative group and each helminth-infected group, and an adjusted *p* (q value) ≤ 0.05 considered statistically significant. In some cases, where potential differences in concentrations of multiple individual bile acids were being examined between experimental groups, multiple Mann–Whitney tests were used, with a 1% false discovery rate approach used to minimize the chances of a type I error.

### Methods statement

All methods were carried out in accordance with relevant guidelines and regulations.

## Results

### Study design

**Fig. 1:**
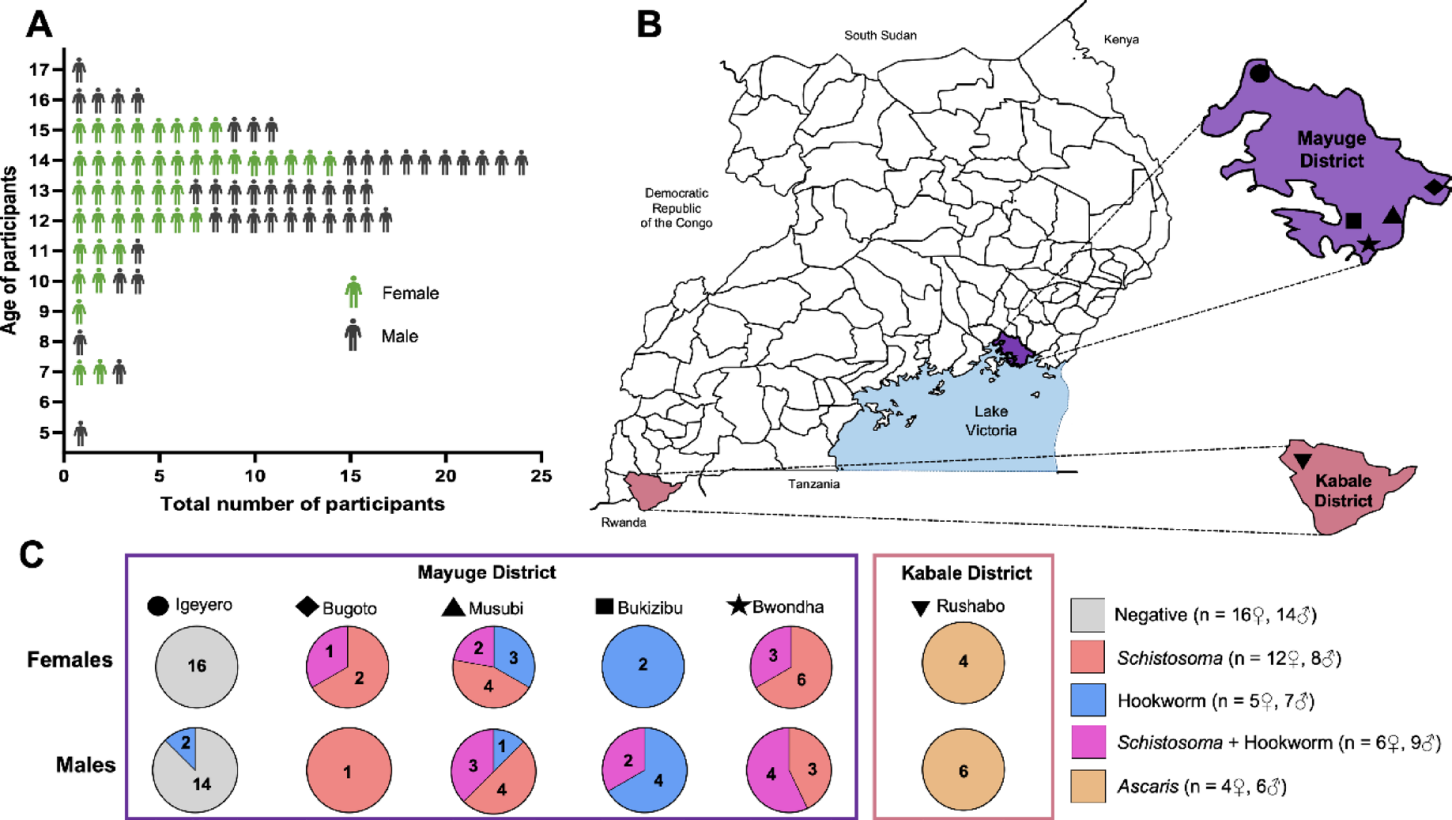
Demographic information showing age, sex, location, and helminth infection status of study participants. (**A**) Age and sex distribution of study participants. (**B**) Location of participants within two districts in Uganda: Mayuge and Kabale, with villages within these districts indicated using different symbols (Igeyero black circle; Bugoto black diamond; Musubi black triangle; Bukizibu black square; Bwondha black star; Rushabo black inverted triangle. (**C**) Number of participants with each helminth infection status are shown separated based on sex, district, and village, and the combined n for each sex and helminth infection status is shown to the right of the panel.

Fecal samples were collected from study participants over a two-day period and each participant’s helminth infection status was determined through the Kato-Katz diagnostic technique. Participants included in this analysis were school-aged children—ranging between 5- and 17-years old at the time of sample collection—with an almost equal distribution of females (n = 43) and males (n = 44) (Fig. 1A). Fecal samples were collected from villages (Igeyero, Bugoto, Musubi, Bukizibu, Bwondha, Rushabo) within two districts in Uganda; Mayuge and Kabale (Fig. 1B). Notably, different regions had varying prevalence of helminth infection or coinfections, with study participants classified as either negative for helminth infection, *Schistosoma*-infected, hookworm-infected, *Schistosoma* and hookworm-coinfected, or *Ascaris*-infected (Fig. 1C).

We assessed the fecal bile acid pool in study participants using targeted mass spectrometry, allowing us to quantify 65 individual bile acids. We did not find any statistically significant differences in the moisture content of fecal samples from those with different helminth infection statuses (Fig. S1), and as such, all bile acid concentrations in this report are presented as nmol/dry mass of feces. Bile acid concentrations per wet fecal masses, as well as raw individual bile acid concentration data for all study participants are included in S1 Data.

Previous work has established that the bile acid pool is influenced by biological sex of the host^18^, and for this reason, we chose to analyze all data from female and male participants separately. Indeed, we noted that within some helminth infection groups, there were differences in total fecal bile acid concentrations between females and males (Fig. S2).

### Helminth infection- and sex-associated differences in concentrations of total and major bile acid subcategories in feces

**Fig. 2:**
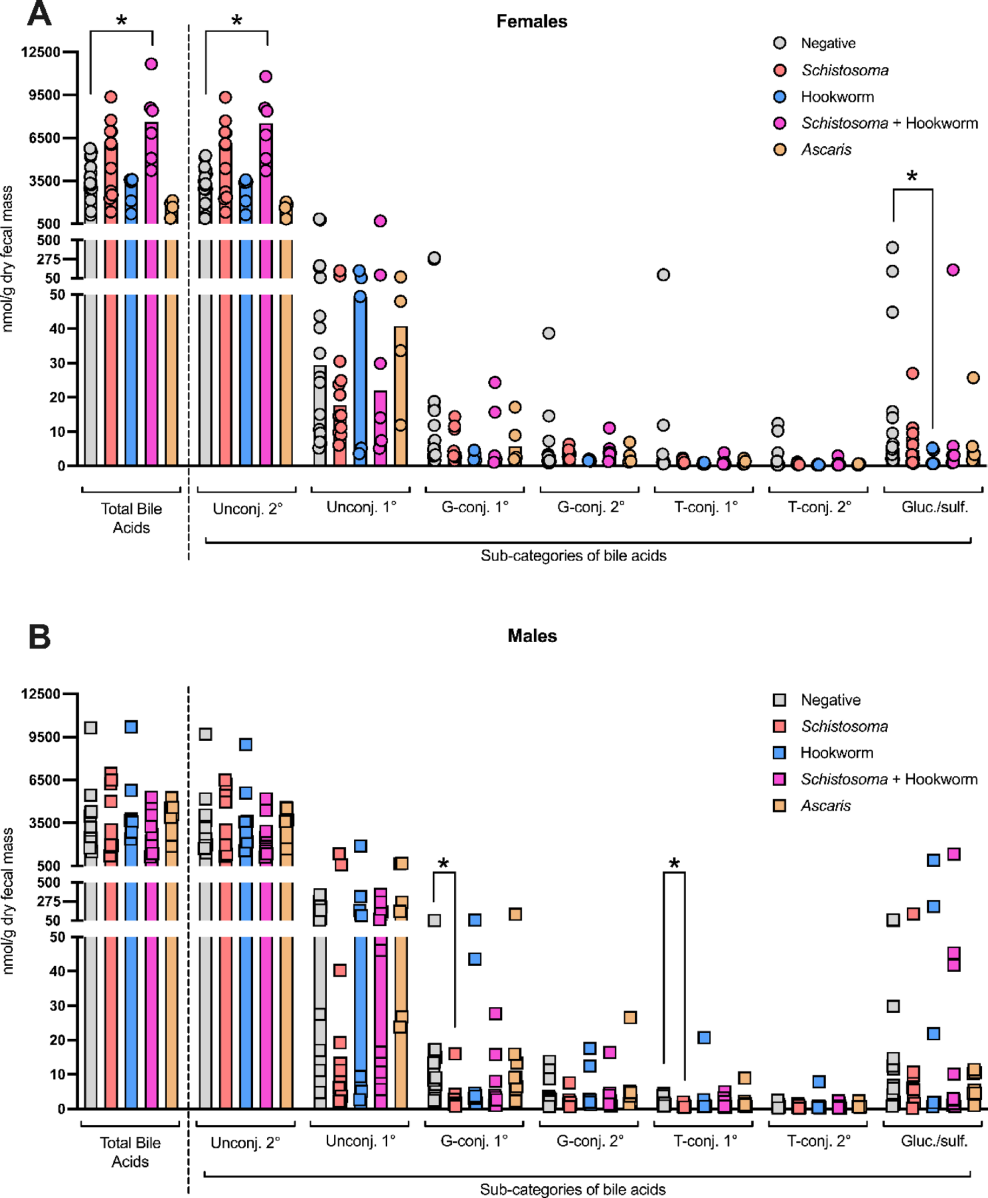
Unconjugated secondary bile acids dominate the fecal bile acid pool, and their concentration is associated with helminth infection status in female individuals. Fecal bile acid concentrations from (**A**) female and (**B**) male individuals with indicated helminth infection statuses were quantified using targeted mass spectrometry and presented as nmol/gram of dry fecal mass. Each data point represents data from an individual and median values for each group are represented by bar heights. Total bile acid concentrations (combined concentrations of 65 individual bile acids assays) are shown to the left of the dotted line, and to the right of the dotted line subcategories of bile acids within the total bile acid pool are presented, from left to right: unconjugated secondary, unconjugated primary, glycine-conjugated primary, glycine conjugated secondary, taurine-conjugated primary, taurine-conjugated secondary and combined gluconated and sulfonated bile acids. For total bile acid data and each bile acid category presented, statistical comparisons between each helminth infection status and the helminth-negative group were made using a Kruskal–Wallis test followed by a Dunn’s multiple comparisons test, and * indicates *p* =  ≤ 0.05.

We evaluated total fecal bile acid concentrations, and the combined concentrations of major subcategories of bile acids in all helminth infection status groups in both sexes (Fig. 2A,B). The bile acid pool composition is known to vary based on the sampling sites within the intestine, and the fecal bile acid pool is distinct and dominated by unconjugated secondary bile acids^19^. In line with these expectations, we found that unconjugated secondary bile acids dominate the fecal bile acid pool (Fig. 2A,B) in all study participants. In females, individuals with a *Schistosoma* and hookworm coinfection had elevated levels of both total bile acids and unconjugated secondary bile acids compared to helminth-negative participants (Fig. 2A). Females infected with a *Schistosoma* infection alone showed trends towards elevated total bile acid and unconjugated secondary bile acids compared to helminth-negative participants, although no statistically significant differences were detected between these groups (Fig. 2A). In males, there were no detectable associations between any helminth infection status and concentrations of total or unconjugated secondary bile acids (Fig. 2B). Of the less abundant fecal bile acid subcategories (unconjugated primary, glycine-conjugated primary, glycine-conjugated secondary, taurine-conjugated primary, taurine-conjugated secondary, gluconated or sulfated), we also noted differing concentrations based on helminth infection status and sex. In males, *Schistosoma* infection was associated with reduced concentrations of conjugated primary bile acids compared to helminth-negative males, and in females, hookworm infection was associated with reduced concentrations of gluconated or sulfated bile acids compared to helminth-negative females (Fig. 2A,B, S1 data).

**Fig. 3:**
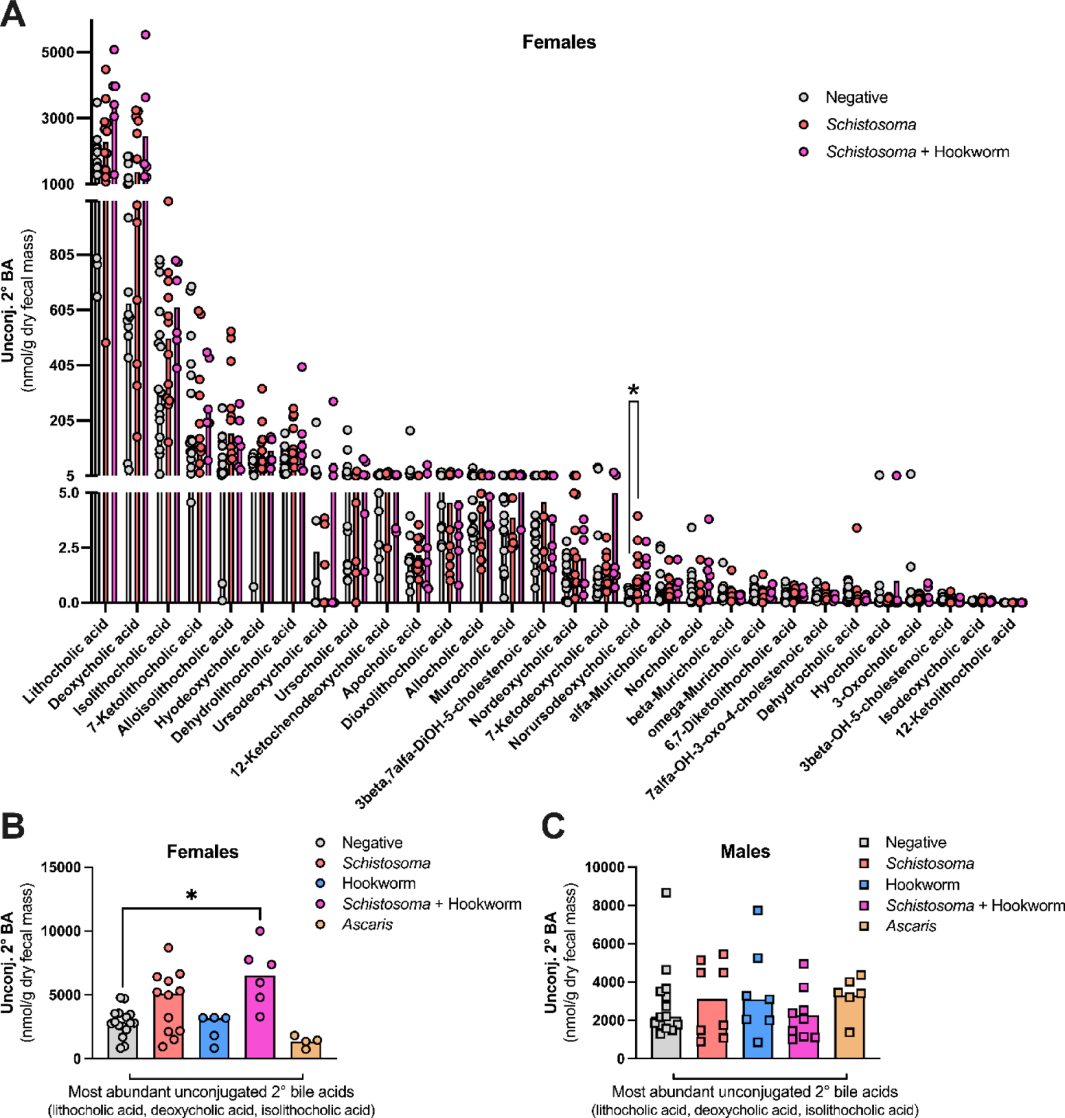
A cumulative increase in the concentration of the three most abundant unconjugated secondary fecal bile acids is associated with *Schistosoma* and hookworm co*-*infection in females. Concentrations of individual unconjugated secondary fecal bile acids were determined through targeted mass spectrometry in individuals with the indicated helminth infection status and presented as nmol/gram of dry fecal mass. Each data point represents data from an individual and median values for each group are represented by bar heights. (**A**) Individual unconjugated secondary fecal bile acid concentrations from females that were either helminth infection negative, *Schistosoma* infected, or *Schistosoma* and hookworm coinfected. Statistical comparisons between helminth-negative and *Schistosoma*-infected, and between helminth-negative and *Schistosoma* and hookworm-coinfected individuals were made for each bile acid using multiple Mann–Whitney tests with a 1% false discovery rate approach to minimize the chances of a type 1 error; q values above 0.01 were considered not statistically significant and * indicates q =  ≤ 0.01. (**B**, **C**) Cumulative concentrations of the three most abundant unconjugated secondary bile acids in feces (lithocholic acid, deoxycholic acid, and isolithocholic acid) are presented as nmol/gram of dry fecal mass in (**B**) females and (**C**) males with the indicated helminth infection status. Statistical comparisons between each helminth infection status and the helminth-negative group were made using a Kruskal–Wallis test followed by a Dunn’s multiple comparisons test, and * indicates *p* =  ≤ 0.05.

Considering the higher concentrations of unconjugated secondary bile acids in the feces of females with a *Schistosoma* and hookworm coinfection compared to helminth-negative females (Fig. 2A), we next examined how the concentration of individual unconjugated secondary bile acids differed between individuals with or without *Schistosoma* infection or coinfection (Fig. 3A). We noted a significant increase in the concentration of the unconjugated secondary bile acid norursodeoxycholic acid in the feces of *Schistosoma*-infected females compared to helminth-negative females (Fig. 3A, S1 data; q value from multiple Mann–Whitney multiple comparisons test = 0.004). A similar trend of increased norursodeoxycholic acid concentrations in *Schistosoma* and hookworm-coinfected females in comparison to helminth-negative females was noted, however this did not reach our threshold for statistical significance (Fig. 3A, S1 data; q value from multiple Mann–Whitney multiple comparisons test = 0.092). However, the increase in this relatively scarce individual unconjugated secondary bile acid was insufficient to explain the phenotype of increased fecal unconjugated secondary bile acid concentrations associated with *Schistosoma* and hookworm coinfection in females. Instead, we noted trending increases in the concentrations of the three most abundant individual unconjugated secondary fecal bile acids (lithocholic acid, deoxycholic acid, and isolithocholic acid) in both *Schistosoma*-infected and *Schistosoma* and hookworm-coinfected females compared to helminth-negative females, which, when examined individually did not reach our threshold for statistical significance (Fig. 3A, S1 data). However, examining the cumulative concentrations of these three abundant unconjugated secondary bile acids revealed that their combined increases were sufficient to explain the phenotype of increased fecal bile acid concentrations in *Schistosoma* and hookworm-coinfected females (Figs. 2A, 3B. Fig. S1 data). We did not detect differences in the cumulative concentrations of the three most abundant unconjugated secondary bile acids or in the concentrations of any individual unconjugated secondary bile acid between helminth-negative and *Schistosoma*-infected or coinfected male participants, nor in individuals of either sex infected with hookworm alone or with *Ascaris* (Fig. 3C, Fig. S3, S1 data).

### Concentrations of low abundance conjugated primary bile acids in feces are associated with helminth infection status and sex

**Fig. 4:**
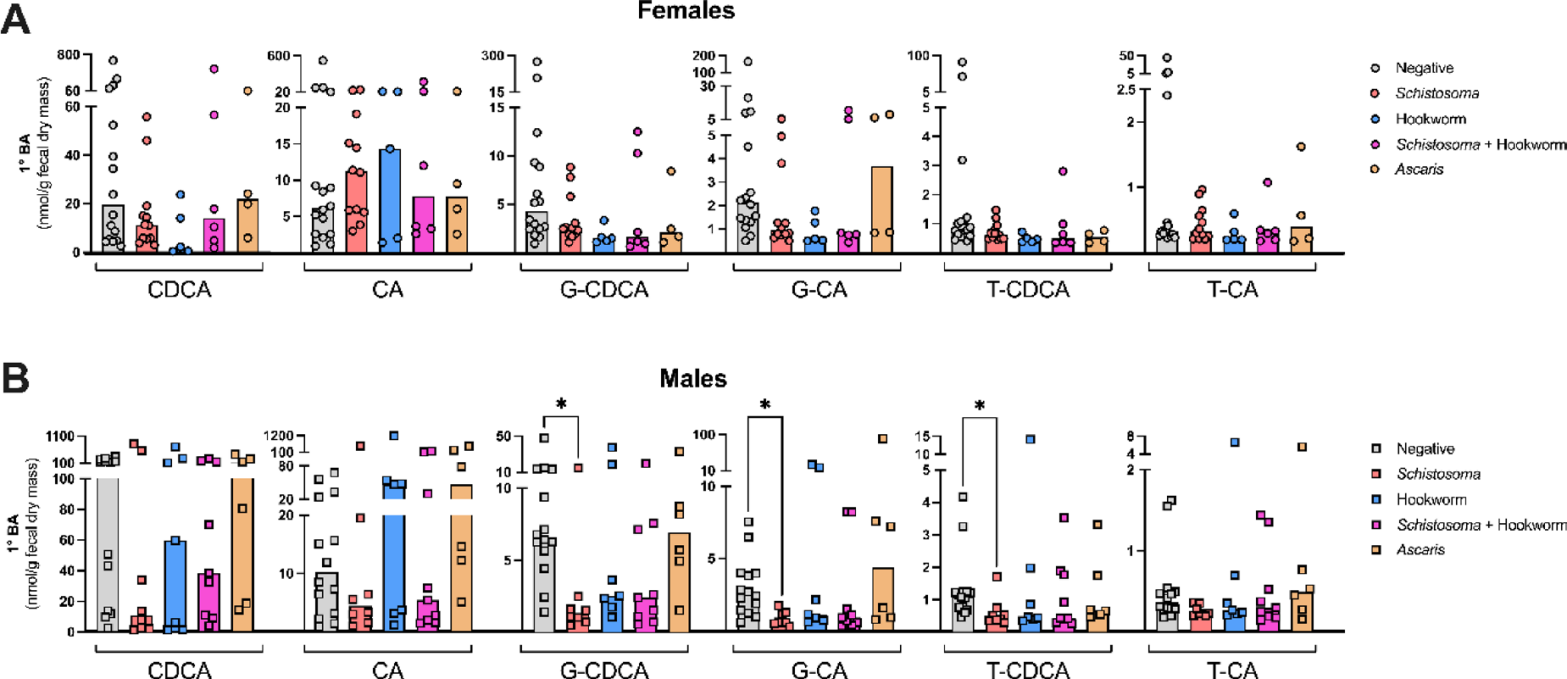
*Schistosoma* infection is associated with a decrease in concentrations of certain conjugated primary bile acids in males. Fecal bile acid concentrations from (**A**) female and (**B**) male individuals with indicated helminth infection statuses were quantified using targeted mass spectrometry and presented as nmol/gram of dry fecal mass. Individual primary bile concentrations are presented, from left to right: unconjugated chenodeoxycholic acid (CDCA), unconjugated cholic acid (CA), glycine-conjugated (G-)CDCA, G-CA, taurine-conjugated (T-)CDCA and T-CA. Each data point represents data from an individual and median values for each group are represented by bar heights. Statistical comparisons between each helminth infection status and the helminth-negative group were made using a Kruskal–Wallis test followed by a Dunn’s multiple comparisons test, and * indicates *p* =  ≤ 0.05.

We next examined the concentrations of the unconjugated primary bile acids (CDCA and CA) and the glycine- or taurine-conjugated primary bile acids in the fecal samples of study participants, which, on average made up less than 5% of the total fecal bile acid pool. In female participants, we did not detect any statistically significant differences in the concentration of any of the unconjugated or conjugated primary bile acids between the different helminth infection statuses (Fig. 4A). However, in male participants, we found that *Schistosoma* infection was associated with a significant decrease in the concentrations of G-CDCA, G-CA, and T-CDCA compared to helminth-negative participants (Fig. 4B).

We further examined which individual bile acids drove the decrease in combined concentrations of gluconated or sulfated bile acids in hookworm-infected females compared to helminth-negative females (Fig. 2A), and found reduced concentrations of two low-abundance sulfated bile acids in hookworm-infected females (glycolithocholic acid 3-sulfate and glycochenodeoxycholic acid 3-sulfate), which did not reach our threshold for statistically significant differences (q values from multiple Mann–Whitney multiple comparisons test = 0.027 and 0.025 respectively; Fig. S4, S1 data); these trends were not apparent in males (Fig. S4, S1 data).

Taken together, our data reveal associations between fecal bile acid concentrations and helminth infection status that appear to be sex-dependent.

## Discussion

To our knowledge, this is the first study to examine associations between helminth infection status and the composition of the fecal bile acid pool in humans. Bile acids regulate many aspects of intestinal physiology, and can play a role in controlling the intestinal microbiota, colonization by microbial pathogens, and regulate intestinal immune responses^7^. As such, it is of interest to understand how colonization with helminths may impact the host bile acid pool. We report associations between the composition and concentration of fecal bile acids and helminth infection status in school-aged children in Uganda. Our study cohort included both female and male children who resided in different districts and villages, and who either had no detectable helminth infection, infections with the soil-transmitted helminths *Ascaris* or hookworms, infections with water-transmitted schistosomes, or coinfections with schistosomes and hookworm. We found notable sex differences in our results: in females but not males that were coinfected with *Schistosoma* and hookworms, total fecal bile acid concentrations were higher than in uninfected individuals, which was driven by increased levels of the most abundant unconjugated secondary bile acids: lithocholic acid, isolithocholic acid (both CDCA derivatives) and deoxycholic acid (a CA derivative). In males but not females, we found reduced levels of certain conjugated primary bile acids associated with *Schistosoma* infection: fecal concentrations of glycine-conjugated CDCA, glycine-conjugated CA, and taurine-conjugated CDCA were lower in male *Schistosoma-*infected individuals compared to helminth-negative males. These findings emphasize the importance of assessing biological sex as a variable when examining potential perturbations to the bile acid pool.

Biological sex is known to impact the composition and total bile acid pool size of mammals: estrogens can impact bile acid synthesis in the liver, as well affecting the enterohepatic circulation of bile acids through impacting transport of bile acids from the intestines to the liver^18^. These sex-based differences in bile acid regulation are evident during steady state conditions, and further, there are notable differences in bile acid regulation between females and males in response to challenges such as dietary interventions^18^. It is probable that sex hormone differences impacting bile acid synthesis/regulation^18^, microbiota compositional/functional differences^20^, response to helminth infection^21^ or a combination of these factors contribute to the sex-based differences in the bile acid pool associated with helminth infections that we noted within our study cohort.

The helminth species identified within this study each have distinct lifecycles within the human host, elicit distinct immune responses at different stages/sites of infection^22^, and each helminth species likely has different impacts on the composition and functional capacity of the bacterial microbiota^23^: all providing opportunities for these infections to differentially disrupt bile acid synthesis, enterohepatic circulation, and the chemical modification of bile acids. In the Mayuge district of Uganda, where all schistosome-infected study participants resided, *Schistosoma mansoni* is the most prevalent schistosome species^24–28^. After penetration of the skin by infectious cercariae, *S. mansoni* parasites migrate in the blood to the liver (the site of primary bile acid synthesis and enterohepatic bile acid ‘recycling’), where they mature, then migrate to mesenteric venules. *S. mansoni* eggs are metabolically active and must migrate across the intestinal wall to be expelled into the feces, however, a large proportion of eggs produced are disseminated by blood flow and ultimately become trapped in the liver, eliciting granuloma formation and ultimately liver fibrosis. In other disease contexts, liver fibrosis has been associated with disruptions to bile acid homeostasis^22^. We speculate that the degree of pathology/chronicity of *S. mansoni* infection would contribute to the degree of disruption to bile acid synthesis and regulation in schistosome-infected individuals. Indeed, in a study assessing circulating metabolite changes in people diagnosed with *S. japonicum* infection, levels of G-CA, G-CDCA, and T-CDCA were higher in the sera of those with evidenced of more advanced-stage schistosomiasis^11^.

The soil-transmitted helminths detected in our study (hookworm and *Ascaris*) enter the human body via the skin and oral route respectively. Parasite larvae migrate through lungs before ultimately establishing infections as adults in the small intestinal lumen. In some cases, migrating larvae are detected at other sites, including the liver, where they have been associated with pathology^29^. When in the intestinal tract, adult worms are directly exposed to the intestinal bile acid pool, as well as the resident microbiota. It is well established that bidirectional relationships exist between the bacterial microbiota and soil-transmitted helminths, with soil-transmitted helminth infections impacting the composition and functional capacity of the bacterial microbiota, and evidence that the microbiota can impact the establishment of helminths within the intestinal tract^30^. Given that the capacity for bile acid modifications is widespread amongst members of the bacterial microbiota^8^, it is reasonable to speculate that enteric helminth infection-driven changes to the bacterial microbiota could mediate disruption to the intestinal bile acid pool^30^. We did not detect associations between concentrations of any of the highly abundant unconjugated secondary bile acids and infection with soil-transmitted helminths, but we did detect changes in levels of some of the lesser abundant sulfated bile acids in hookworm-infected females, possibly driven by changes to the microbiota during infection. We did not have sufficient fecal sample available to assess bacterial microbiota compositions alongside bile acid analyses in this study, and so we were unable to explore potential correlations between particular microbial groups and bile acid abundances within this data set.

In non-human animal experimental settings, helminth infections have been shown to modify host metabolites concentrations, including accumulating evidence of perturbations to the bile acid^31^. For example, fecal metabolite disruptions during either a *S. mansoni, S. japonicum* or *S. mekongi* infection in female mice suggested enhancement of primary bile acid synthesis pathways during infection, although this report did not include targeted approaches to quantify specific bile acids in feces^12^. Previous work from our group has demonstrated that infection of both female and male mice with the small intestine dwelling roundworm *H. polygyrus bakeri* disrupts the bile acid pool at the site of infection: the proximal small intestine, including reductions in concentrations of T-CDCA and T-α-MCA (both primary bile acids in mice), and the conjugated secondary bile acids taurodeoxycholic acid and taurolithocholic^13^. It is possible that localized disruption to the bile acid pool in proximal regions of the intestinal tract are not evident when examining bile acids excreted in feces, which may partly explain why we did not detect major disruption to the bile acid pool in fecal samples from hookworm- or *Ascaris*-infected people in this present study.

Alterations to the host bile acid pool during helminth infection have the potential to impact immune responses either directed towards the helminth itself, or, to impact bystander immune responses in helminth infected people, as previous work has demonstrated the immunomodulatory capabilities of bile acids signaling through host bile acid receptors^32,33^. Indeed, during *H. polygyrus bakeri* infection in mice, there is reduced signalling through the bile acid receptor, farnesoid X receptor (FXR)^13^, and in various contexts, modulated FXR signalling has been shown to impact host immune responses^34,35^. There is also the possibility that changes to the bile acid pool may also have direct consequences for helminths, perhaps serving as an energy source or signaling cue: bile has been shown to influence larvae migration of roundworms^36^, and certain bile acids have been shown to stimulate oviposition in schistosomes^37^. While the impacts of helminth infection-derived changes to the bile acid pool on human health remain to be elucidated, utilization of rodent models to explore associations between helminth infections and bile acids noted in human studies may reveal causal relationships and mechanisms of interactions.

Several limitations should be noted about our study. Firstly, we must acknowledge that the relatively low number of participants examined- particularly of those who presented with *Ascaris* or hookworm infections- reduces our ability to statistically detect subtle changes to the fecal bile acid composition. Secondly, by nature of the distribution of helminth infections in Uganda, different regions and villages are dominated by different helminth infection statuses. While this distribution presents a valuable opportunity to study the impact of different helminth infections/coinfections on the bile acid pool, it also means that the area of residence of study participants becomes a confounding variable that may impact the bile acid pool independently of helminth infection status. For example, dietary and/or climate differences between regions of Uganda likely also impact the bile acid pool. Due to the relatively low number of participants examined, we did not have sufficient numbers to examine the impact of helminth burdens within each helminth infection status on the fecal bile acid pool, although we would speculate that both helminth burden and the chronicity of infection would influence the impact of infection on the bile acid pool. It is possible that some study participants that we classed as negative for helminth infection had a low burden infection that was not detectable by our diagnostic methods. In addition, it is possible that some study participants had co-infections with other pathogens prevalent in Uganda, such as *Plasmodium* spp*.*^38,39^, human immunodeficiency virus^40^, and/or hepatitis B^41^. These potential co-infections have been associated with impacts on liver function^42,43^ and gut microbiota alterations^44^. Finally, the fecal bile acid pool is distinct from the bile acid pool along the intestinal tract^19^, and therefore our study was unable to assess whether perturbations to the bile acid pool along the intestinal tract occurred during helminth infection. Despite these limitations, our hope is that future investigations can build on this work, including larger cohorts to determine if similar or additional associations between different helminth infections and the fecal bile acid pool are detected, including expanding these studies to different world regions where helminth infections are endemic. It would be of interest to examine how the composition of bile acids that re-circulate in the blood may be impacted by different helminth infections. Investigating the impact of deworming on the fecal bile acid pool would also be of interest, to see if/when helminth-infection associated perturbations to the bile acid pool revert following helminth clearance. Although there are notable differences in the bile acid pool and bile acid regulation between humans and mice^45^, we believe rodent studies will be valuable in determining causal relationships between helminth infection status, the bile acid pool at various sites along the intestinal tract, and potential functional impacts of bile acid disruptions on host health.

In summary, our study provides, to our knowledge, the first insight into the interplay between helminth infection status and fecal bile acid composition in humans. Advancing our understanding of host-parasite interactions may allow us to develop strategies to improve the health of those living in helminth endemic areas, and/or identify mechanisms to promote intestinal health more generally.

## Electronic supplementary material

Below is the link to the electronic supplementary material.

Supplementary Material 1

Supplementary Material 2

## Data Availability

All data generated or analysed during this study are included in this article (and its Supplementary Information files).
