# Supplementary material for: Associations between helminth infection status and the composition and concentration of fecal bile acids in school-age children in Uganda
Source: Sci Rep. 2025 Jul 15;15:25509. doi: 10.1038/s41598-025-11170-z (PMC12264275; doi:10.1038/s41598-025-11170-z)
Supplement: Supplementary file 2 — Supplementary Material 2 [file 41598_2025_11170_MOESM2_ESM.docx]

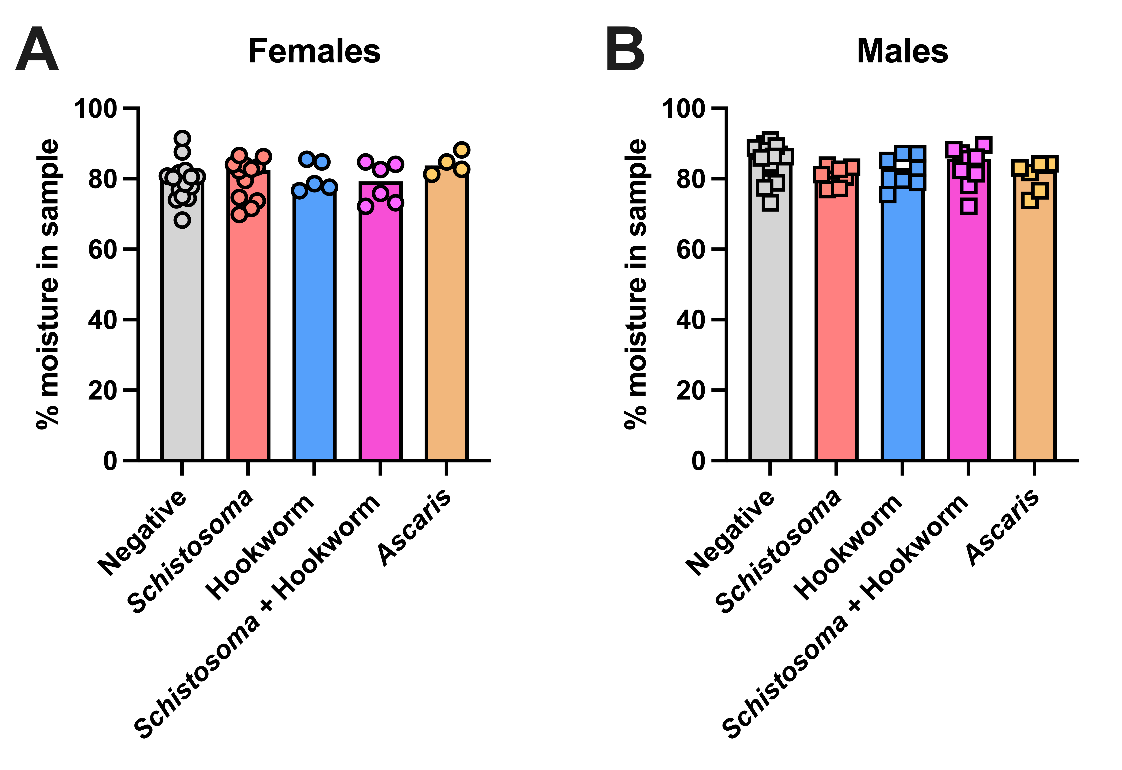


**S1 Fig. Fecal moisture content was consistent between individuals with different helminth infection statuses.** Fecal samples from study participants were weighed before and after lyophilization, to give wet and dry fecal masses, respectively. Percent fecal moisture content was determined for (A) female and (B) male participants by calculating the % of mass lost after lyophilization ([wet mass-dry mass]/wet mass*100). Each data point represents values for an individual participant and bars heights are at median values for each group. Statistical comparisons between each helminth infection status and the helminth-negative group were made using a Kruskal-Wallis test followed by a Dunn’s multiple comparisons test, and no statistically significant differences were detected.


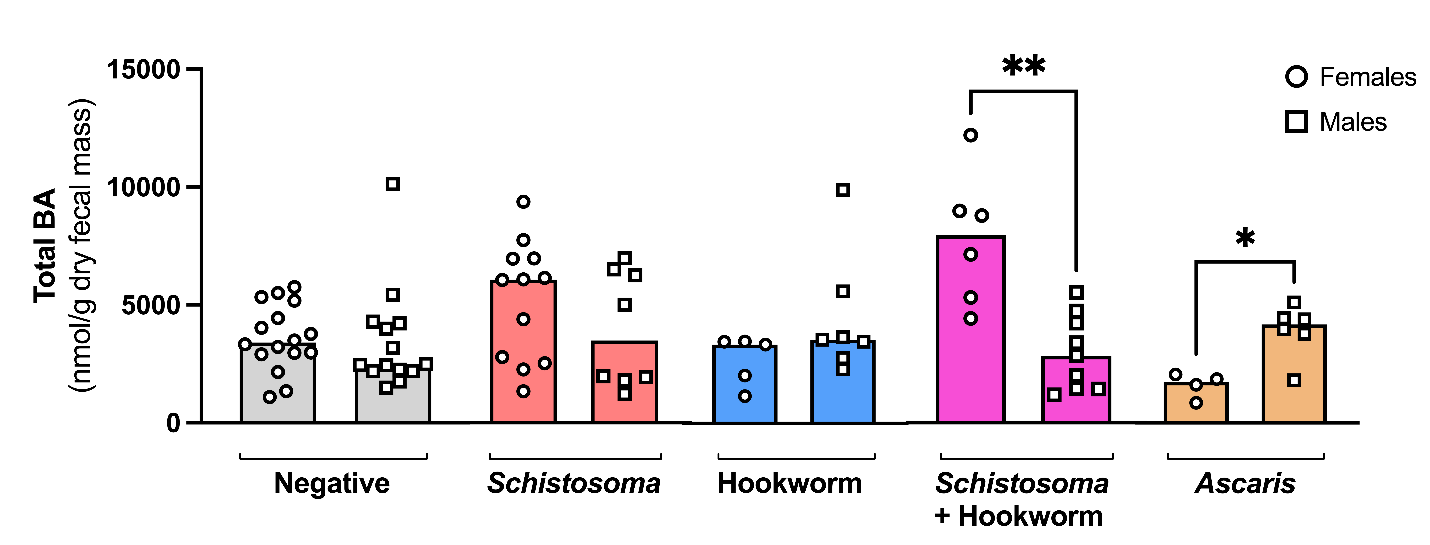


**S2 Fig. Biological sex of participants impacts total fecal bile acid concentrations.** Fecal bile acid concentrations were determined through targeted mass spectrometry. The combined total concentration of all 65 bile acids assayed is presented as nmol/gram of dry fecal mass. Each data point represents data from an individual and median values for each group are represented by bar heights. Mann-Whitney tests were done between females and males for each helminth infection status, with * indicating p = ≤ 0.05 and ** indicating p = ≤ 0.01.


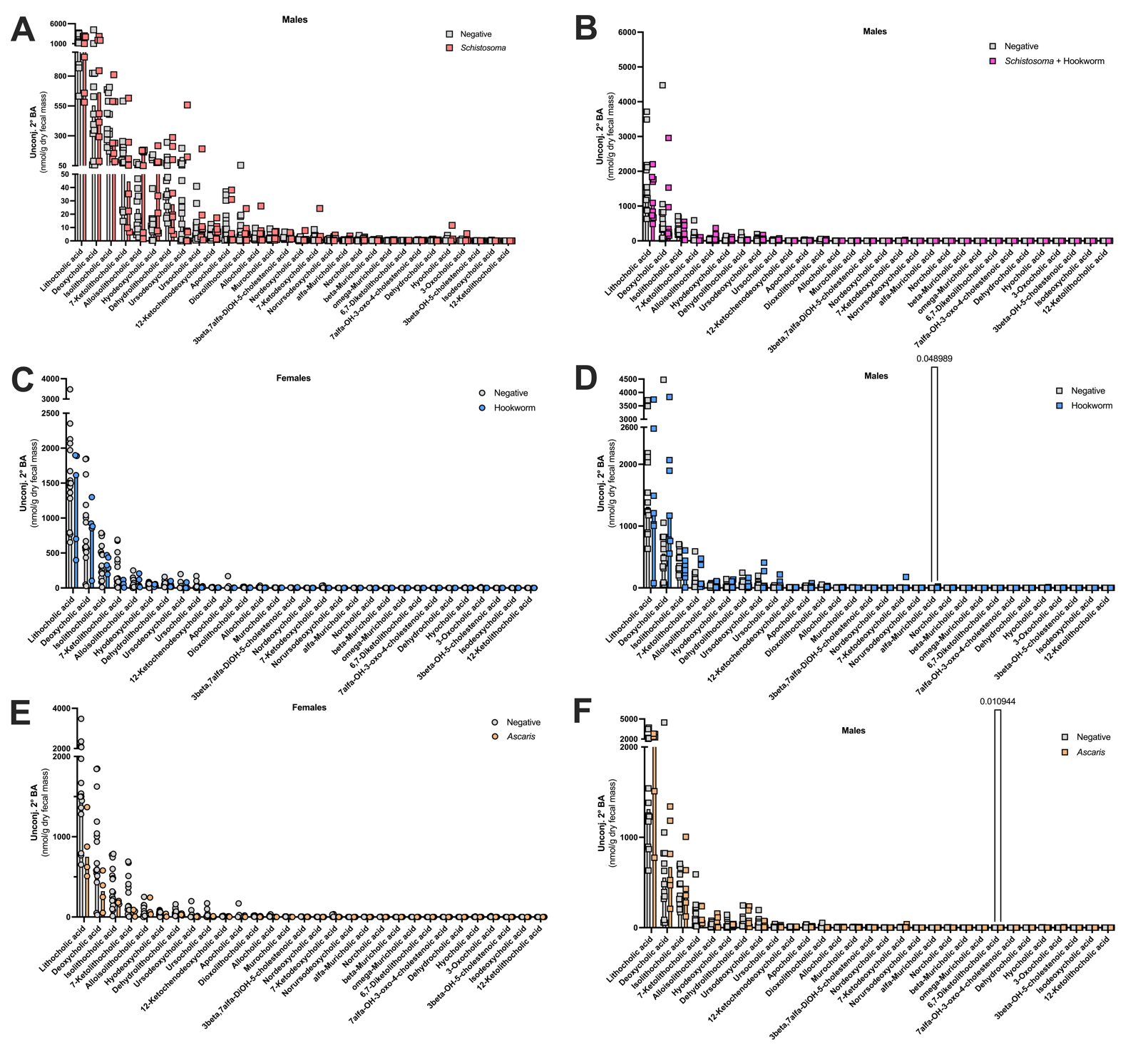


**S3 Fig.** **No statistically significant differences in the concentrations of individual unconjugated secondary fecal bile acids in *Schistosoma* or *Schistosoma* + hookworm-coinfected males or in either sex infected with hookworm or *Ascaris* in comparison to helminth-negative individuals.** Fecal bile acids were determined through targeted mass spectrometry and presented as nmol/gram of dry fecal mass. Data from helminth-negative male individuals is presented in comparison to data from (A) *Schistosoma-*infected males and (B) *Schistosoma* + hookworm-coinfected males. (C) Data from helminth-negative females is presented in comparison to data from hookworm-infected females. (D) Data from helminth-negative males is presented in comparison to data from hookworm-infected males. (E) Data from helminth-negative females is presented in comparison to data from *Ascaris*-infected females. (F) Data from helminth-negative males is presented in comparison to data from *Ascaris-*infected males. Each data point represents results obtained from one individual and median values for each group are represented by bar heights. For data shown in each graph, statistical comparisons were made for each bile acid between the helminth-negative and helminth-infected group using multiple Mann-Whitney tests with a 1% false discovery rate approach to minimize the chances of a type 1 error; q values ≤ 0.05 are displayed on graphs; all q values were above 0.01 and thus not deemed statistically significant.


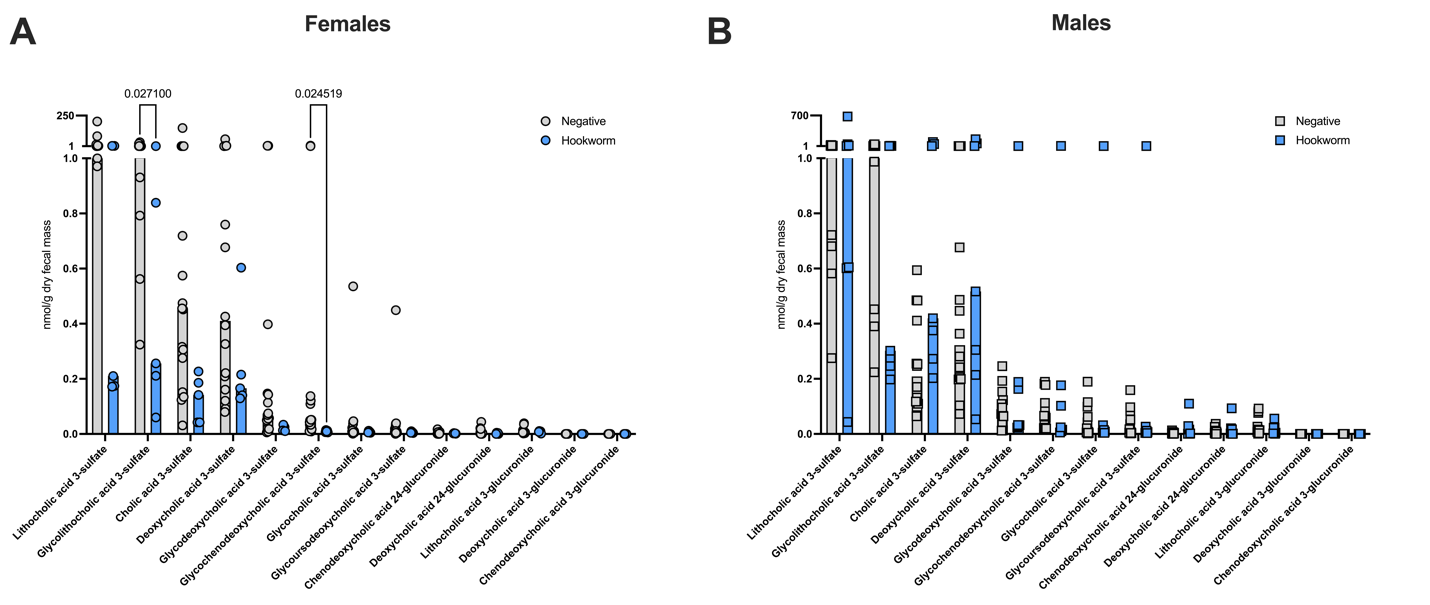


**S4 Fig. Concentrations of certain individual sulfated bile acids trend towards decreased concentrations in the feces of hookworm-infected females, in comparison to helminth-negative females.** Fecal bile acids were determined through targeted mass spectrometry and presented as nmol/gram of dry fecal mass. Concentrations of individual gluconated or sulfonated bile acids in helminth-negative individuals in comparison to hookworm-infected individuals are presented for (A) females and (B) males. Each data point represents results obtained from one individual and median values for each group are represented as bars on graphs. Statistical comparisons were made for each bile acid between the helminth-negative and helminth-infected group using multiple Mann-Whitney tests with a 1% false discovery rate approach to minimize the chances of a type 1 error; q values ≤ 0.05 are displayed on graphs; all q values were above 0.01 and thus not deemed statistically significant.
